# Improving Oxidation Resistance by Addition of Boron in FeNi-Based Superalloy

**DOI:** 10.3390/ma19173628

**Published:** 2026-08-26

**Authors:** Cham Il Kim, Won Tae Kim, Eun Soo Park, Do Hyang Kim, Heon Kang

**Affiliations:** 1 Department of Materials Science and Engineering, Yonsei University, Yonsei-ro 50, Seodaemun-gu, Seoul 03722, Republic of Korea; ckadlf@kitech.re.kr; 2 Korea Institute of Industrial Technology, Siheung-si 15014, Gyeonggi-do, Republic of Korea; 3 Department of Optical Engineering, Cheongju University, 298 Daeseong-ro, Cheongwon-gu, Cheongju-si 28503, Chungcheongbuk-do, Republic of Korea; wontae@cju.ac.kr; 4 Department of Materials Science and Engineering, Research Institute of Advanced Materials & Institute of Engineering Research, Seoul National University, Seoul 08826, Republic of Korea; espark@snu.ac.kr

**Keywords:** FeNi-based superalloy, boron addition, oxidation resistance, creep rupture life, interfacial boron segregation

## Abstract

**Highlights:**

**What are the main findings?**

- B0 developed a porous oxide scale exceeding 5 μm after 100 h.
- B0.1 and B0.2 retained uniform oxide layers of approximately 417 and 405 nm.
- GD-MS revealed boron segregation at the oxide/matrix interface.

**What are the implications of the main findings?**

- Boron is associated with improved oxide-scale stability.
- A small boron addition is sufficient to stabilize the oxide layer.
- Interfacial segregation is key to oxidation-resistant superalloy design.

**Abstract:**

This study investigates the effect of trace boron addition on the oxidation resistance of FeNi-based superalloys based on Incoloy 706. Although boron is known to improve high-temperature mechanical properties, its role in oxidation remains controversial because it can either enhance or degrade oxide-scale stability depending on its distribution. To clarify this effect, boron-free, 0.1 at% B-added, and 0.2 at% B-added alloys were fabricated and oxidized at 1023 K under isothermal conditions. In the boron-free alloy, a thin Cr- and Al-rich oxide layer initially formed but rapidly thickened after 50 h, developing into a porous Fe_2_O_3_/Cr_2_O_3_ scale exceeding 5 μm after 100 h. In contrast, the boron-containing alloys maintained thin and uniform spinel-rich Fe–Cr mixed oxide scales of approximately 400 nm after 100 h, with no significant porosity. TGA/DSC analysis showed gradual weight gain without exothermic peaks in B0.1 and B0.2, whereas B0 exhibited accelerated oxidation after approximately 75 h. Glow discharge analysis of B0.1 revealed boron segregation at the oxide/matrix interface. These results suggest that interfacial boron segregation is associated with improved oxide-scale stability and may contribute to enhanced oxidation behavior.

## 1. Introduction

Superalloys are widely used in turbine engines of aircraft due to their high strength, creep resistance, and resistance to fatigue crack growth at elevated temperatures [1,2]. Increasing the turbine inlet temperature can lead to better engine performance, which has driven the development of alloys with superior high-temperature properties. Therefore, studying the oxidation behavior of superalloys is essential in enhancing high-temperature properties.

There are various reports on the oxidation behavior of Fe-based alloys. Sun et al. investigated the corrosion of Fe–25Ni–15Cr under the combined effects of high-temperature oxidation. The results indicated that the high-temperature oxidation rate was significantly accelerated by the precipitated salts and corrosion products after field exposure [3]. Additionally, Elliott et al. [4] examined the oxidation behavior of Incoloy 800 alloy at high temperatures (1073 K) in an environment containing HCl gas in moist air. Hussain et al. [5] provided important insights into how Incoloy 825 reacts to high-temperature oxidation and spalling under various atmospheric conditions, particularly regarding its oxidation resistance at elevated temperatures. Zhang et al. [6] reported that the Ni–Fe-based superalloy, Ni–32Fe–17Cr, exhibits superior oxidation resistance compared to Cr20Ni80 at temperatures above 1373 K, with an external oxide layer composed of Cr_2_O_3_ and NiAl_2_O_4_ protecting it from further oxidation, and demonstrated higher strength under the same operating conditions. Khalid et al. [7] compared the oxidation tendencies of two Fe-Ni-based superalloys, Incoloy 800H and Incoloy 825, at high temperatures, concluding that oxidation resistance is superior when a Cr-rich oxide layer is formed instead of a spinel oxide layer.

Ni-based or FeNi-based superalloys generally benefit from doping with trace amounts of boron. Boron strengthens grain boundaries, thereby improving creep life, rupture strength, and tensile ductility at elevated temperatures [8,9,10]. Klein et al. [11,12] discovered that in addition to enhancing mechanical properties, boron addition significantly increases the oxidation resistance of Co-based superalloys at 1073 K. However, Zhao et al. [13] also reported that the addition of boron to Ni-based forged superalloys with high Al and Ti contents during homogenization treatment affects oxidation behavior by forming boron-rich oxide layers, which disrupt the continuity of the Cr_2_O_3_ layer and, through their decomposition, make the Cr_2_O_3_ layer more porous, significantly degrading its protective properties and consequently reducing oxidation resistance.

Klein et al. [11] found that boron accumulates in the internal oxide layer of Co-based superalloys. Jalowicka et al. [14] suggested that after 20 h of oxidation at 1323 K, a semi-continuous BCrO_3_ layer formed at the base of the Cr_2_O_3_ scale on the conventionally cast Ni-based superalloy Rene 80 due to the diffusion of boron toward the alloy surface. Recently, Kontis et al. [15] reported that an intermediate oxide with the stoichiometry of Al_4_B_2_O_9_ formed in the boron-containing Ni-based superalloy STAL15-CC after 10,000 h of oxidation at 1173 K, with boron segregating at the grain boundaries of the Al_4_B_2_O_9_ phase. These studies analyzed the distribution of boron within the oxide layers during the stable oxidation stage. However, how the distribution and form of boron change as oxidation progresses remains unclear. While several researchers have studied the distribution of boron after oxidation, the mechanism by which boron affects the oxidation resistance of superalloys is not well understood, and analysis of boron’s impact on the oxidation behavior of FeNi-based superalloys is limited. Clarifying this aspect will help understand the mechanism by which boron influences high-temperature oxidation resistance in superalloys. Therefore, this study investigates the oxidation behavior of FeNi-based superalloys based on Incoloy 706 by comparing boron-free, 0.1 at% B-added, and 0.2 at% B-added alloys. In particular, this work aims to clarify not only the beneficial effect of boron addition but also whether increasing boron concentration influences oxide growth kinetics and oxide-scale stability. 

## 2. Materials and Methods

The composition of the alloys used in the present study is given in Table 1. Alloy samples with a thickness of 5 mm were prepared by arc-melting metals with a purity > 99.9 at% in an Ar atmosphere and then casting into a suction mold. The cast samples were homogenized at 1423 K for 12 h and hot-rolled into 2 mm-thick sheets at temperatures of 1423 K. Then the sheet samples were solution-treated at 1423 K for 30 min to dissolve unwanted precipitates formed during hot rolling. Then the plate samples were aged at 1023 K for 20 h to precipitate γ′-Ni_3_Ti and γ″-Ni_3_Nb phases. The isothermal oxidation tests for SEM/EDS and XRD characterization were conducted at 1023 K in a box furnace under static laboratory air for 20, 50, and 100 h.

Microstructural characteristics, including the phase composition before and after the oxidation test, were analyzed using a field-emission scanning electron microscope (FE-SEM; JSM-7001F, JEOL Ltd., Akishima, Tokyo, Japan) equipped with an energy-dispersive spectrometer (EDS; EDAX, EDAX Inc., Mahwah, NJ, USA). To investigate the thermal oxidation behavior under isothermal conditions, oxidation tests were conducted at 1023 K for 100 h in dry air using a thermogravimetric analyzer (TGA) and differential scanning calorimetry (DSC) system (TGA/DSC; TGA/DSC 1, Mettler-Toledo AG, Greifensee, Switzerland). 

The mass change measured by TGA was normalized by the total exposed surface area of each specimen and expressed in mg/cm^2^. The total exposed surface areas used for normalization were 0.271408, 0.245466, and 0.254672 cm^2^ for the B0, B0.1, and B0.2 specimens, respectively. The total exposed surface area was calculated from the measured specimen dimensions assuming a rectangular geometry, A=2(ab+bc+ca), where a, b and c represent the specimen dimensions. The normalized mass gain was calculated as ∆m/A, where ∆m is the change in specimen mass measured by TGA. For analysis of the isothermal oxidation behavior, the mass measured at the beginning of the isothermal stage at 1023 K was defined as the reference mass (∆m = 0), thereby excluding the apparent mass change occurring during the heating stage. The normalized mass change was calculated as ∆m/A, where ∆m is the mass change relative to the beginning of the isothermal stage and *A* is the total exposed surface area of the specimen. To quantitatively compare the mass-change behavior during isothermal oxidation, the data were divided into four time intervals (0–20, 20–50, 50–75, and 75–100 h), and the mass-change rate for each interval was determined by linear regression.

The specimens were heated from room temperature to 1023 K at a heating rate of 40 K/min and subsequently held isothermally for the designated oxidation time. Prior to oxidation testing, the specimen surfaces were mechanically ground using SiC abrasive papers up to #2000 grit, ultrasonically cleaned in acetone, and dried in air before testing. The data were analyzed using STARe Software V18.00 (Mettler-Toledo AG, Greifensee, Switzerland). Analysis of the oxide layer and identification of the location and role of boron were conducted using a Glow Discharge Mass Spectrometer (GD-MS; ELEMENT GD, Thermo Fisher Scientific, Bremen, Germany) and a Glow Discharge Spectrometer (GD-OES; GDS850A, LECO Corporation, St. Joseph, MI, USA). GD-OES measurements were conducted at 1000 V, 30 W, and 30 mA, with a measurement area of 4 mm in diameter. The acquisition rate was 10 points/s. Data collected during the first 0.02 s were excluded because of discharge stabilization. The sputtering-time data were converted to depth using the depth-calibration function of the GD-OES analysis system with a depth-conversion calibration factor of 0.85. Based on the acquisition time and the corresponding converted depth values, the effective sputtering rate over the analyzed region was approximately 3.5–4.0 nm/s. The time-to-depth conversion was not strictly linear because the depth increment varied with the analyzed composition. The resulting depth scale is expressed in μm after excluding the initial unstable discharge period (0–0.02 s). The depth profiles were obtained up to approximately 3 μm. The oxide layer and the boron distribution were analyzed using GD-MS (ELEMENT GD, Thermo Fisher Scientific, Bremen, Germany), with a measurement area of 4 mm in diameter. The measurements were conducted in pulse mode with a pulse duration of 100 µs, a pulse frequency of 2 kHz, and a discharge voltage of 750 V. A total of 2000 cycles were analyzed. Additionally, since the measurement is not based on depth distance, the sputtering depth was converted proportionally to the observed depth through OM and the measurement time. 

For the post-creep oxidation analysis, specimens were sectioned immediately adjacent to the fracture plane after creep rupture testing at 1023 K under an applied stress of 320 MPa. The transverse direction (TD) cross-sections were then mounted, mechanically ground, and polished for SEM/EDS analysis.

## 3. Results and Discussion

### 3.1. Oxide Scale Morphology and Elemental Distribution After Isothermal Oxidation

**Figure 1:**
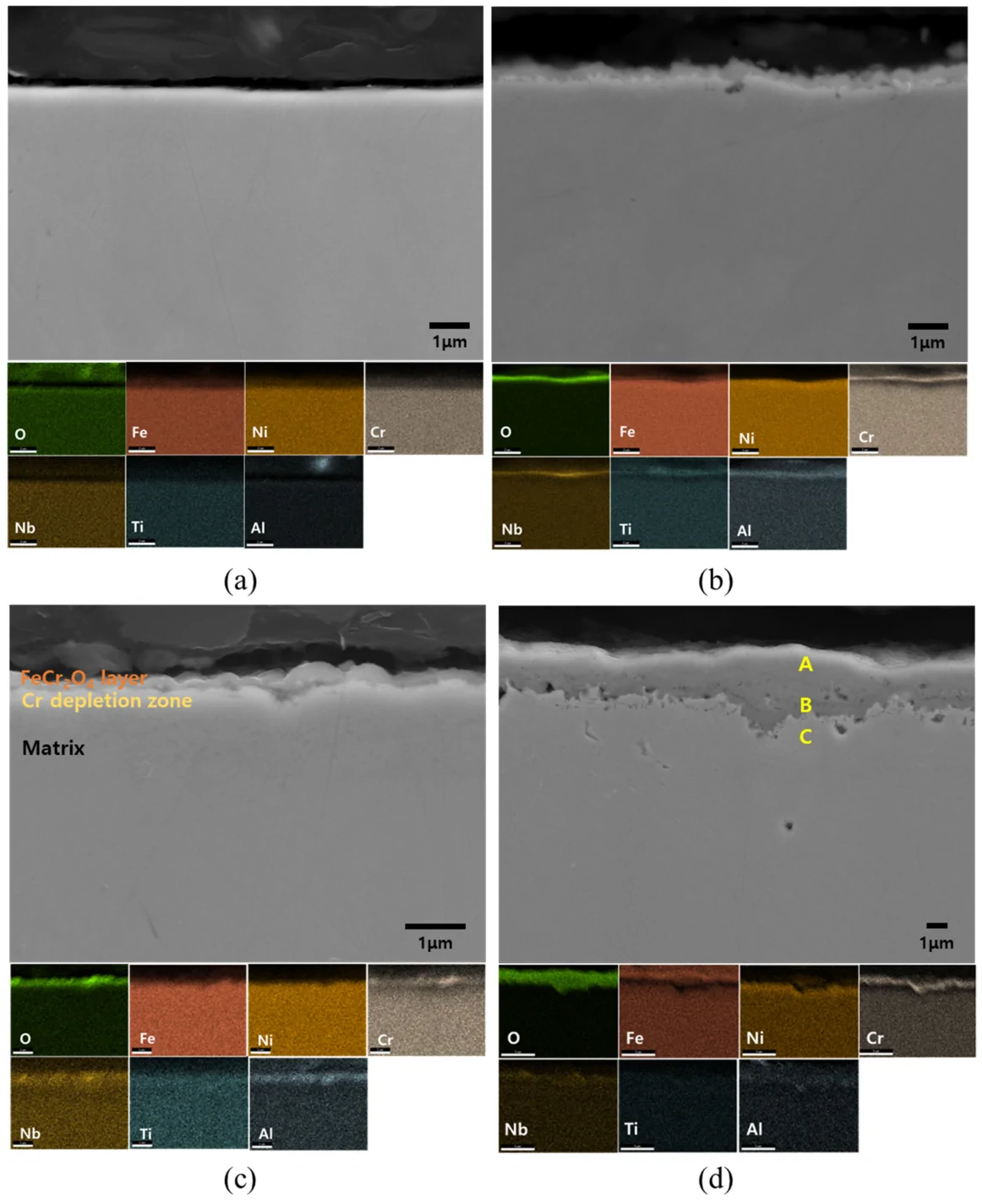
Results of SEM and EDS mapping for compositional elements obtained from the B0 alloy after oxidation: (**a**) before oxidation and after (**b**) 20 h, (**c**) 50 h, (**d**) 100 h. In the EDS maps, O, Fe, Ni, Cr, Nb, Ti, and Al are represented by green, red, yellow, gray, brown, blue, and dark blue, respectively. A, B, and C in (**d**) indicate the oxide layer, Cr-depleted region, and matrix, respectively.

Figure 1 shows the cross-sectional SEM images and EDS composition mapping results obtained from the oxidation layers of the B0 alloy after oxidation at 1023 K for 20 h, 50 h, and 100 h. Oxidation was conducted in air. The oxide layer after 20 h was uniform and thin, with a thickness of approximately 361 nm. After 50 h of oxidation, the oxide layer thickness increased slightly to approximately 381 nm. Unlike the 20 h sample, some uneven porous structures were observed under the oxide layer. After 100 h of oxidation, the oxide layer became significantly thicker, reaching approximately 5.4 μm, and exhibited an irregular thickness distribution, with a porous structure underneath the oxide layer. According to the EDS mapping results, the oxide layer after 20 h was rich in Cr, Nb, Ti, and Al, while the oxide layer after 50 h was rich in Cr, Nb, and Al. In the 100 h oxidation sample, the oxide layer was divided into an Fe-rich layer with high Cr and Nb content and underneath the Al-rich layer. A Cr-depleted zone was commonly observed underneath the oxide layer in all cases. Notably, after 100 h of oxidation, localized voids and porous features were observed beneath the oxide layer within the Cr-depleted region, suggesting degradation associated with prolonged oxidation. 

**Figure 2:**
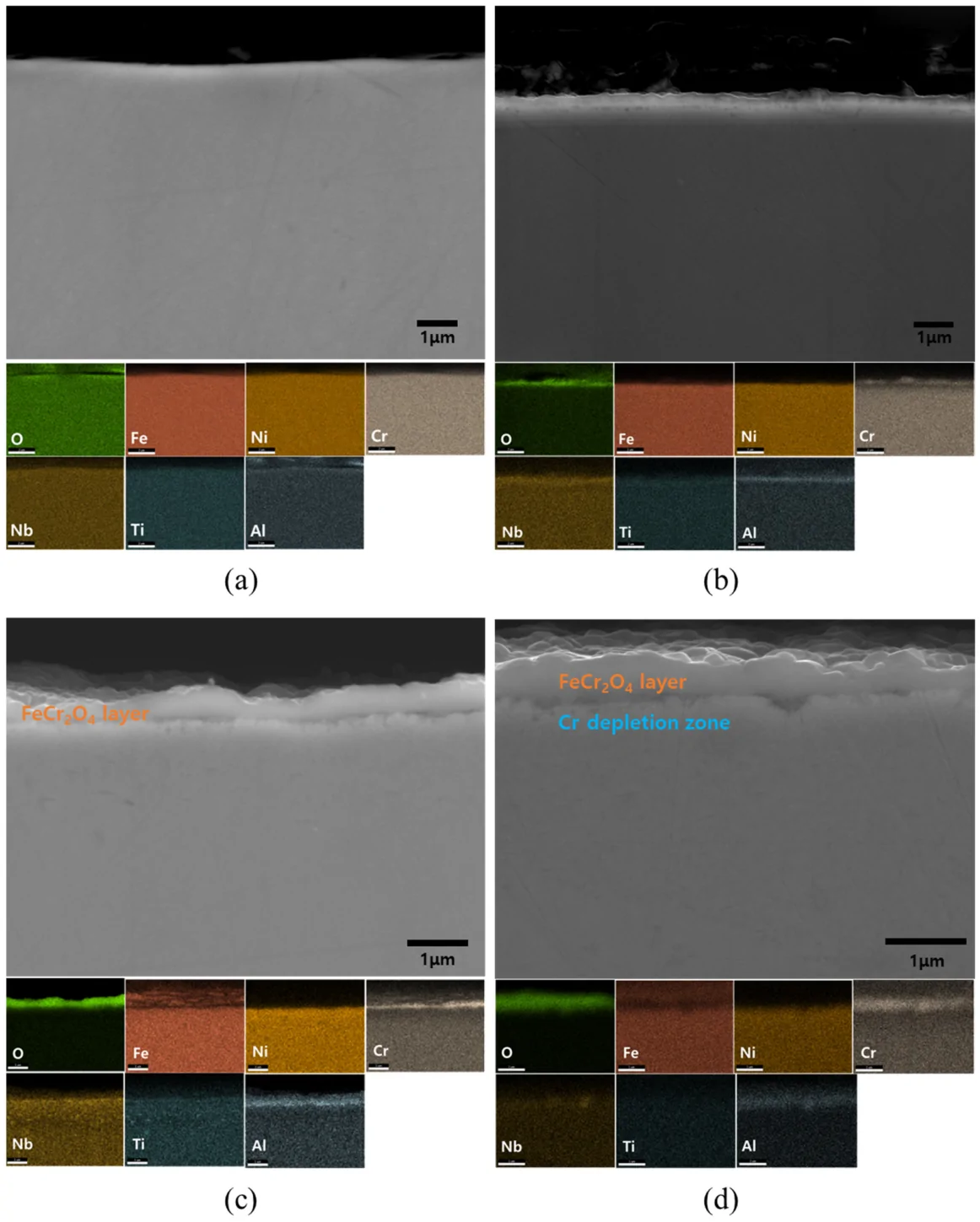
Results of SEM and EDS mapping for compositional elements obtained from the B0.1 alloy after oxidation: (**a**) before oxidation and after (**b**) 20 h, (**c**) 50 h, (**d**) 100 h. In the EDS maps, O, Fe, Ni, Cr, Nb, Ti, and Al are represented by green, red, yellow, gray, brown, blue, and dark blue, respectively.

Figure 2 shows the cross-sectional SEM images and EDS composition mapping results obtained from the oxidation layers of the B0.1 alloy after oxidation at 1023 K for 20 h, 50 h, and 100 h. The B0.1 alloy formed an oxide layer with a thickness of approximately 357 nm after 20 h and approximately 374 nm after 50 h of oxidation, exhibiting a similar trend to the B0 alloy. However, unlike the B0 alloy, the oxide layer in the B0.1 alloy remained stable even after 100 h of oxidation. The oxide layers formed after 20 and 50 h were relatively uniform, with thicknesses of approximately 357 and 374 nm, respectively. After 100 h of oxidation, the oxide layer remained almost unchanged, at approximately 417 nm. Unlike the B0 alloy, a porous structure was not observed underneath the oxide layer even after 50 h of oxidation. EDS analysis showed local compositional variations in the oxide layer with oxidation time. Nevertheless, Fe and Cr remained the major metallic constituents of the oxide scale during prolonged oxidation, while the Ti content was relatively low after 50 and 100 h. Despite these compositional variations, the oxide scale maintained a thin and continuous morphology up to 100 h.

**Figure 3:**
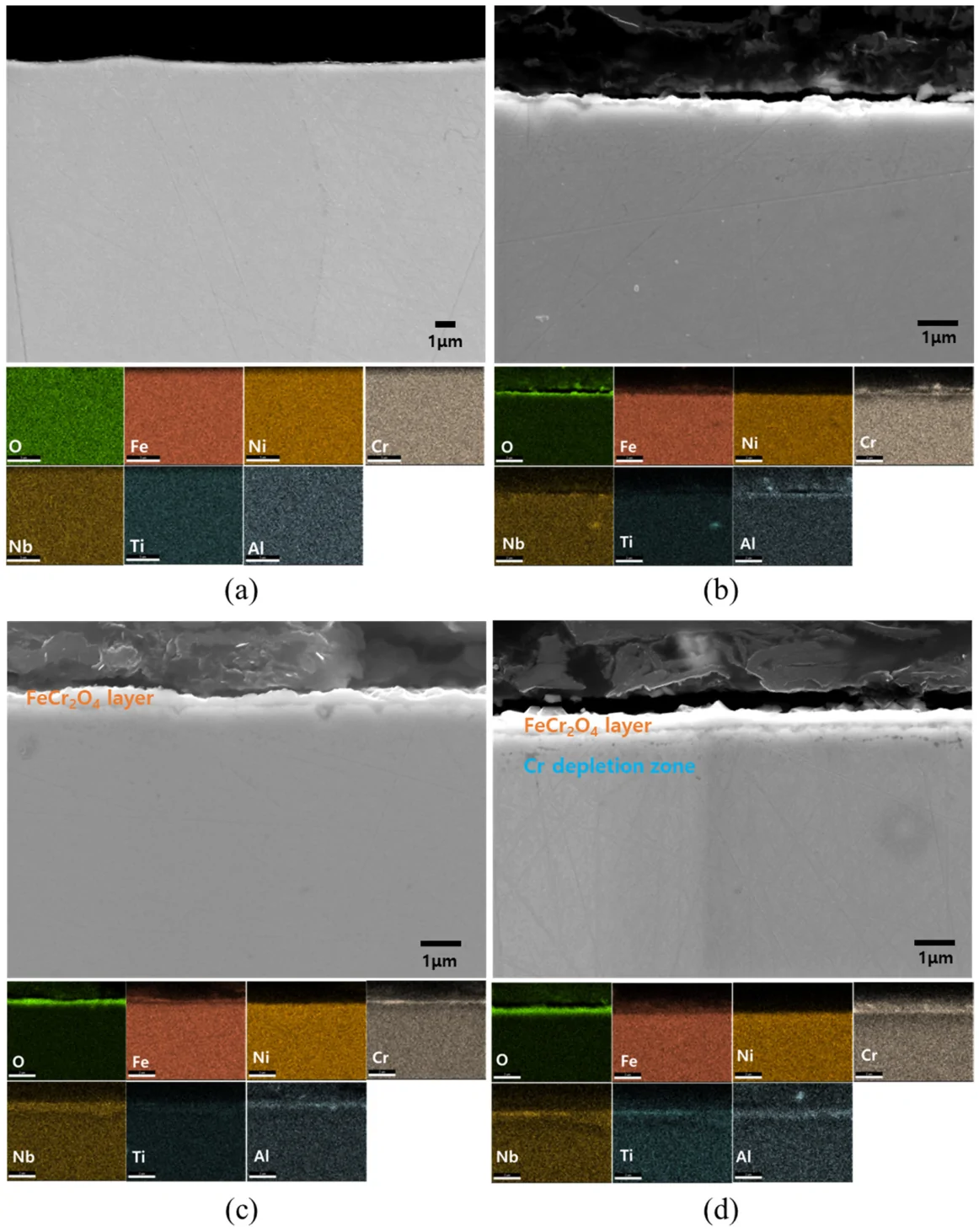
SEM results obtained from the B0.2 alloy after oxidation: (**a**) before oxidation and after (**b**) 20 h, (**c**) 50 h, (**d**) 100 h. In the EDS maps, O, Fe, Ni, Cr, Nb, Ti, and Al are represented by green, red, yellow, gray, brown, blue, and dark blue, respectively.

Additionally, in Figure 3, cross-sectional SEM images and EDS composition mapping results obtained from the oxidation layers of the B0.2 alloy after oxidation at 1023 K for 20 h, 50 h, and 100 h are shown. The B0.2 alloy formed an oxide layer with a thickness of approximately 179 nm after 20 h, 321 nm after 50 h, and 405 nm after 100 h of oxidation. While it exhibited better oxidation resistance than the B0.1 alloy up to 20 h of oxidation, a similar trend was observed as oxidation continued up to 100 h. EDS analysis also showed local compositional variations in the oxide layer with oxidation time. Fe and Cr remained the major metallic constituents of the oxide scale, although their relative concentrations varied during prolonged oxidation. Despite these compositional variations, the oxide scale maintained a thin and continuous morphology up to 100 h, similar to that observed for the B0.1 alloy.

**Figure 4:**
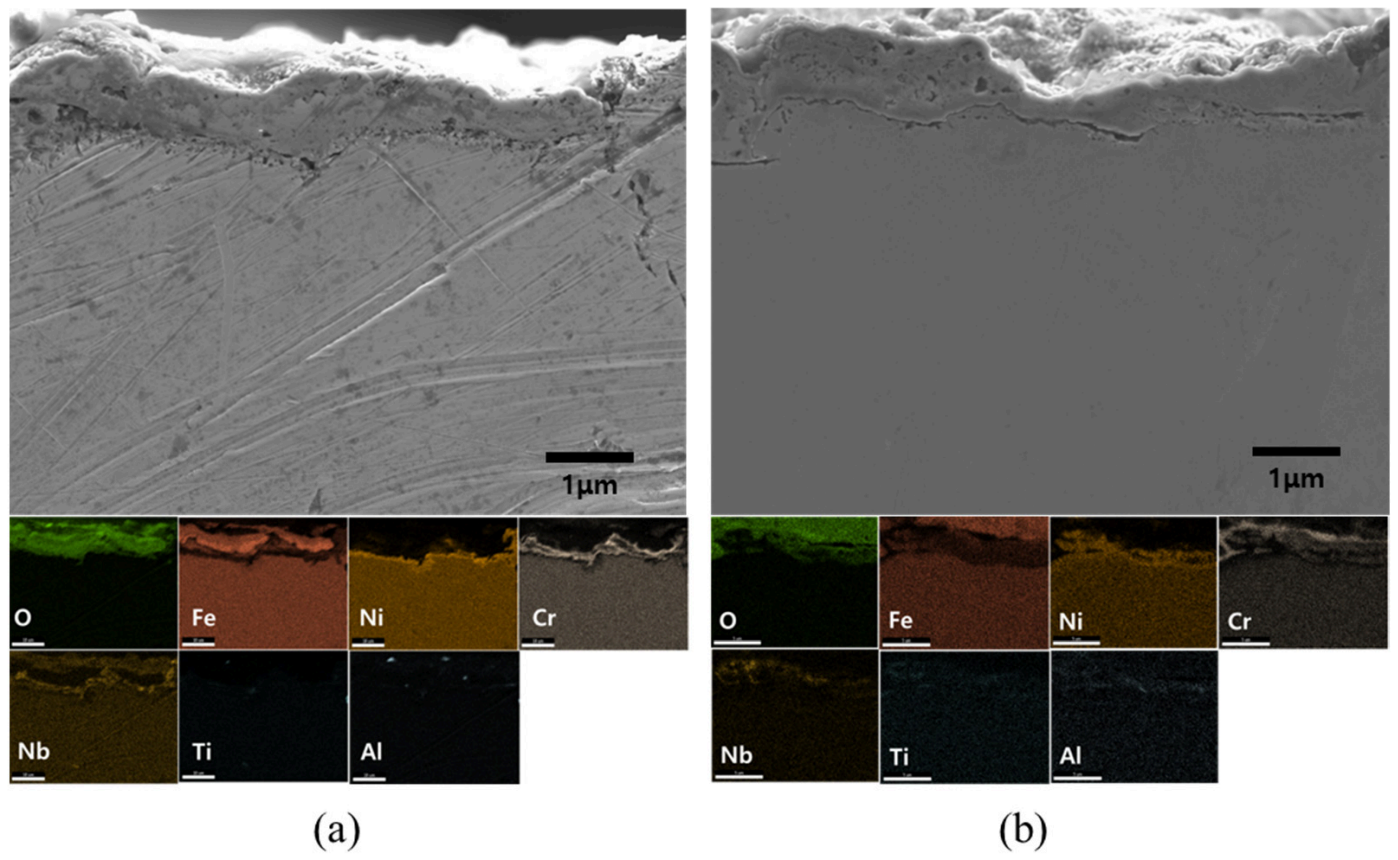
Results of SEM and EDS mapping for compositional elements obtained from the TD (transverse direction) plane after creep failure: (**a**) B0 (320 MPa / 1023 K, failure after 72.78 h), (**b**) B0.1 (320 MPa/1023 K, failure after 167.64 h).

Figure 4 shows cross-sectional SEM images obtained from the transverse direction (TD) surface immediately adjacent to the fracture plane after creep rupture tests conducted at 1023 K under an applied stress of 320 MPa. Since the B0.2 alloy exhibited oxidation behavior comparable to that of the B0.1 alloy during isothermal oxidation, including similar oxide thicknesses, SEM morphologies, XRD patterns, and TGA/DSC responses, the B0.1 alloy was selected as the representative boron-containing alloy for the post-creep oxidation analysis. Figure 4a shows the oxide layer of the B0 alloy after the creep test at 1023 K under a load of 320 MPa (fracture of the sample occurred after 72.78 h). SEM analysis revealed that significant oxidation proceeded on the TD side near the fractured surface, where the load was highly concentrated. SEM analysis further indicated that oxidation was significantly accelerated between 50 h and 100 h, as the initially formed oxide layer could not withstand further oxidation. The more severe oxidation observed after creep rupture is considered to result from the combined effects of prolonged exposure at high temperatures and the applied creep stress, leading to greater oxide-scale development than under unstressed isothermal oxidation. Previous studies have reported that stress-induced oxidation can accelerate the process, and this explains the more severe oxidation compared to unstressed samples [3,17,18,19]. Additionally, the internal structure became more porous. Figure 4b shows the oxide layer of the B0.1 alloy after the creep test at 1023 K under a load of 320 MPa (Fracture of the sample occurred after 167.64 h). The oxide layer thickness was similar to that of the B0 alloy after 72.78 h, but the B0.1 alloy underwent a longer oxidation process, demonstrating that the results suggest improved thermal stability of the oxide layer.

The improved stability of the oxide layer is expected to reduce oxidation-induced degradation during long-term creep exposure. Consequently, the boron-containing alloy maintained oxide-scale integrity for a substantially longer exposure time before rupture, which is consistent with its superior creep rupture life compared with the boron-free alloy. This observation is also consistent with our previous study, in which boron segregation effectively suppressed recrystallization during creep and significantly prolonged the creep rupture life of the same FeNi-based superalloy [16]. Although creep strength is also influenced by microstructural factors, the improved oxidation resistance is considered to have contributed to the prolonged rupture life. Finally, Table 2 summarizes the thickness of the oxide layer. Additionally, the EDS results of the oxide layer for each oxidation time are summarized in Table 3, Table 4 and Table 5. The combined SEM-EDS results indicate that Fe–Cr mixed oxides formed in all alloys, although their morphology and relative composition differed substantially. For the B0 alloy after 100 h oxidation, three different local regions (A–C) were analyzed because the oxide layer became heterogeneous. In the B0 alloy, prolonged oxidation resulted in a heterogeneous and thick Fe-rich oxide scale containing locally separated Fe-rich and Cr-rich regions. In contrast, the B0.1 and B0.2 alloys maintained thin and relatively uniform Fe–Cr mixed oxide scales. When considered together with the XRD results, the oxide scales formed on the boron-containing alloys are consistent with continuous spinel-rich Fe–Cr oxide layers with possible minor contributions from Fe_2_O_3_ and Cr_2_O_3_. Ti-rich oxides were locally detected during the initial stage of oxidation. With increasing oxidation time, the continuous growth of the Fe–Cr mixed oxide scale became dominant, while the Ti-rich oxides remained as minor or locally distributed phases beneath or within the scale, making them less distinguishable by SEM-EDS after prolonged oxidation.

The detailed EDS compositions of the B0, B0.1, and B0.2 alloys at different oxidation times are presented in Table 3, Table 4 and Table 5, respectively.

**Figure 5:**
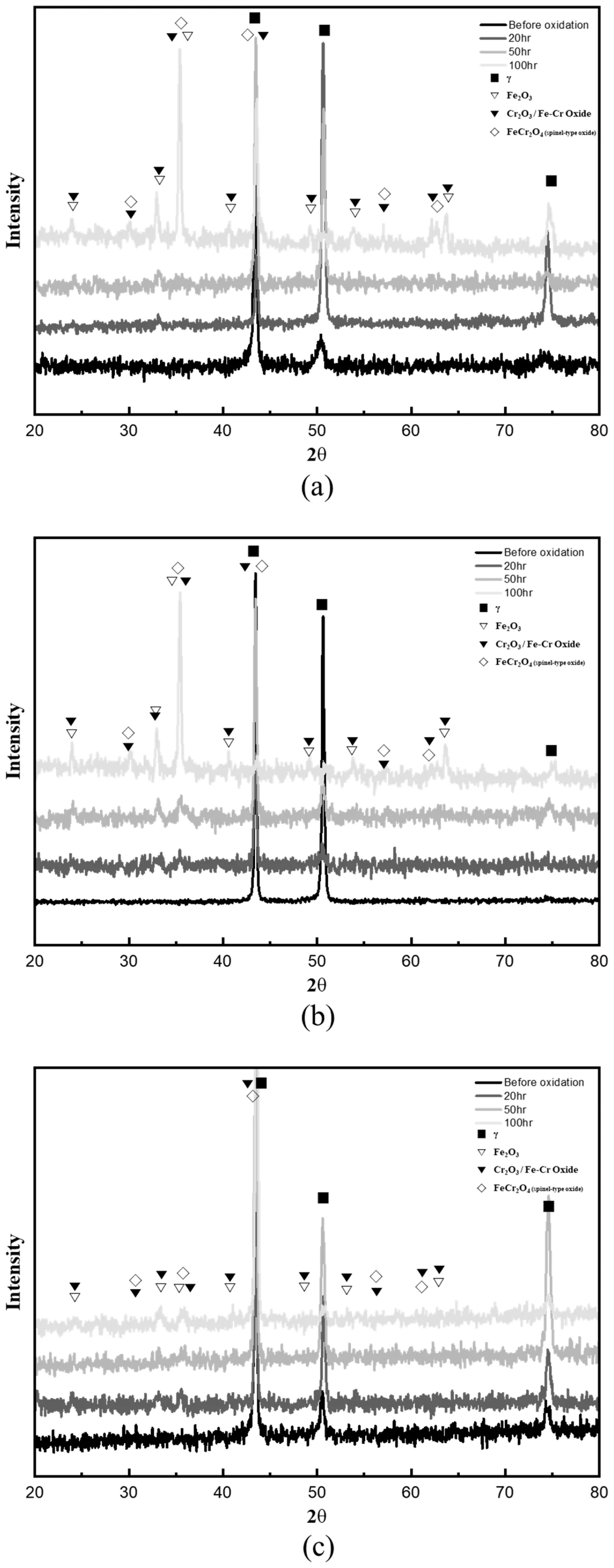
XRD results obtained from (**a**) B0, (**b**) B0.1 and (**c**) B0.2 alloys after oxidation at 0, 20, 50, 100 h. (The diffraction peaks were compared with the ICDD Powder Diffraction File reference patterns for α-Fe_2_O_3_ (PDF No. 33-0664), Cr_2_O_3_ (PDF No. 38-1479), and FeCr_2_O_4_ (spinel-type oxide) (PDF No. 34-0140). Because of substantial peak overlap among the Fe-Cr oxide phases and the γ matrix, the phase assignments are interpreted as tentative).

Figure 5 presents the XRD patterns of the B0, B0.1, and B0.2 alloys before oxidation and after 20, 50, and 100 h of oxidation. During the early and intermediate oxidation stages (20 and 50 h), the diffraction patterns remained largely dominated by the γ-matrix peaks, while oxide-related reflections became more distinguishable with increasing oxidation time, particularly in the B0 alloy. After 100 h of oxidation, the B0 alloy exhibited several additional diffraction peaks attributable primarily to α-Fe_2_O_3_, with possible contributions from Cr_2_O_3_ and FeCr_2_O_4_-type spinel oxides. The oxide scale in B0 was therefore interpreted as a heterogeneous mixture of Fe-rich oxides and Fe-Cr oxides. In comparison, the diffraction patterns of the B0.1 and B0.2 alloys remained dominated by the γ-matrix reflections because of their substantially thinner oxide scales. Nevertheless, the weak oxide reflections, together with the Fe- and Cr-rich compositions observed by SEM-EDS, are consistent with the formation of spinel-rich Fe-Cr mixed oxide scales with an FeCr_2_O_4_-type structure. Minor contributions from α-Fe_2_O_3_ and Cr_2_O_3_ cannot be excluded because several reflections overlap with one another and with the γ-matrix peaks. The similar XRD, SEM, and EDS responses of B0.1 and B0.2 indicate that the two boron-containing alloys formed comparable types of thin and continuous protective oxide scales.

### 3.2. Thermal Oxidation Behavior Under Isothermal Conditions

**Figure 6:**
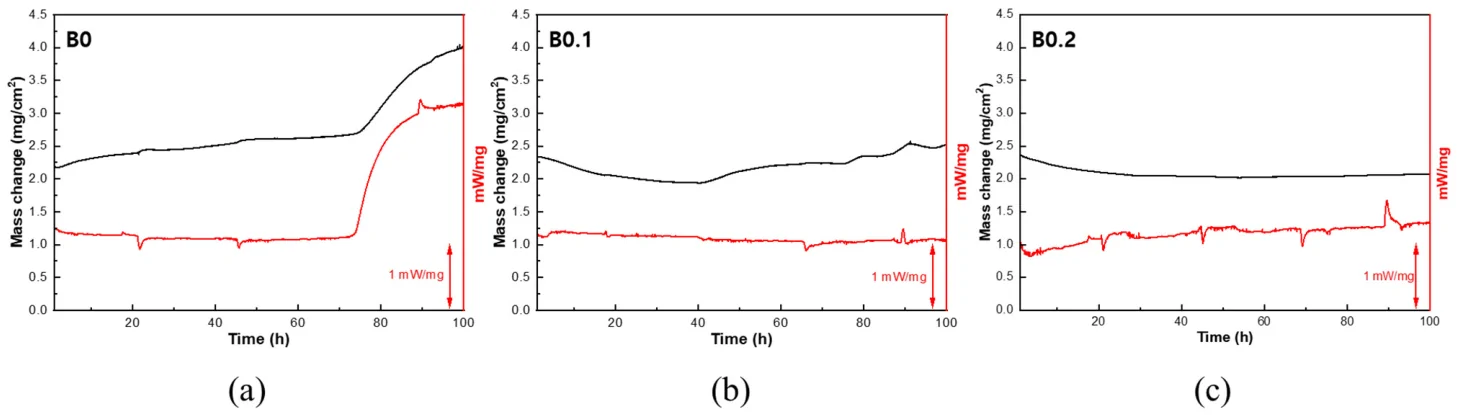
TGA and DSC results of (**a**) B0, (**b**) B0.1, and (**c**) B0.2 alloys during 100 h of isothermal testing in a dry air atmosphere at 1023 K.

Figure 6 shows the TGA and DSC analysis results for the B0, B0.1 and B0.2 alloys. The TGA analysis was conducted for 100 h at 1023 K under isothermal conditions, following a temperature ramp of 40 K/min in a dry air atmosphere. For quantitative evaluation of the isothermal oxidation behavior, the beginning of the isothermal stage at 1023 K was defined as the reference point (∆m = 0), excluding the apparent mass change during the heating stage. The mass-change rates obtained by linear regression over four time intervals are summarized in Table 6. During the early isothermal stage, apparent negative mass-change rates were observed for the B0.1 and B0.2 alloys, particularly for B0.2. These initial negative values are considered to reflect stabilization of the TGA baseline rather than oxidation-induced mass loss and were therefore not interpreted as oxidation kinetics. After 50 h, both boron-containing alloys exhibited positive but relatively low mass-change rates. Most notably, during 75–100 h, the mass-change rate of B0 increased markedly to 0.05201 mg cm^−2^ h^−1^, whereas those of B0.1 and B0.2 remained substantially lower at 0.00246 and 0.00715 mg cm^−2^ h^−1^, respectively. This pronounced increase in the B0 alloy was accompanied by the exothermic peak observed in the DSC curve at approximately 74.5 h, indicating accelerated oxidation during prolonged exposure. It should be noted that the TGA measurements and the specimens used for SEM characterization were obtained under different oxidation environments. The TGA measurements were conducted under a controlled flowing dry-air atmosphere, whereas the specimens for SEM characterization were oxidized in a box furnace under static laboratory air; therefore, the absolute TGA mass change was not directly correlated with the local oxide-scale thickness measured by SEM. Nevertheless, both analyses showed a consistent qualitative trend, with pronounced late-stage oxidation in B0 and comparatively stable oxidation behavior in the boron-containing alloys. To describe the time-dependent mass-change behavior under the present isothermal condition, the normalized mass-change data can be fitted using the following equation:

(1) $$∆m/A=kt^{n}$$

where Δ*m*/*A* is the normalized mass change (mg cm^−2^), *k* is a fitting constant at 1023 K, *t* is the oxidation time, and n is the time exponent. The fitting curves for each data set are shown in Figure 6, and the corresponding time exponent values are presented in Table 7. It is important to note that these trends are based on a limited experimental data set. As shown in Figure 6c, the B0.2 alloy did not exhibit the pronounced increase in mass change observed for the B0 alloy during prolonged oxidation. After 50 h, the B0.2 alloy showed a relatively low positive mass-change rate, and no distinct exothermic peak associated with accelerated oxidation was observed in the DSC curve. This behavior is consistent with the SEM observations, which showed that the oxide scale of B0.2 remained thin and continuous up to 100 h. Although the mass-change behavior of B0.1 and B0.2 was not identical, both boron-containing alloys showed substantially lower mass-change rates than B0 during the final stage of oxidation. Considering that B0.1 and B0.2 also exhibited similar oxide thicknesses after 100 h, increasing the boron content from 0.1 to 0.2 at% did not result in a further pronounced improvement in long-term oxide-scale stability under the present oxidation conditions.

### 3.3. Analysis of the Oxide Layer Using GD-MS and GDS for Identification of the Location and Role of Boron

**Figure 7:**
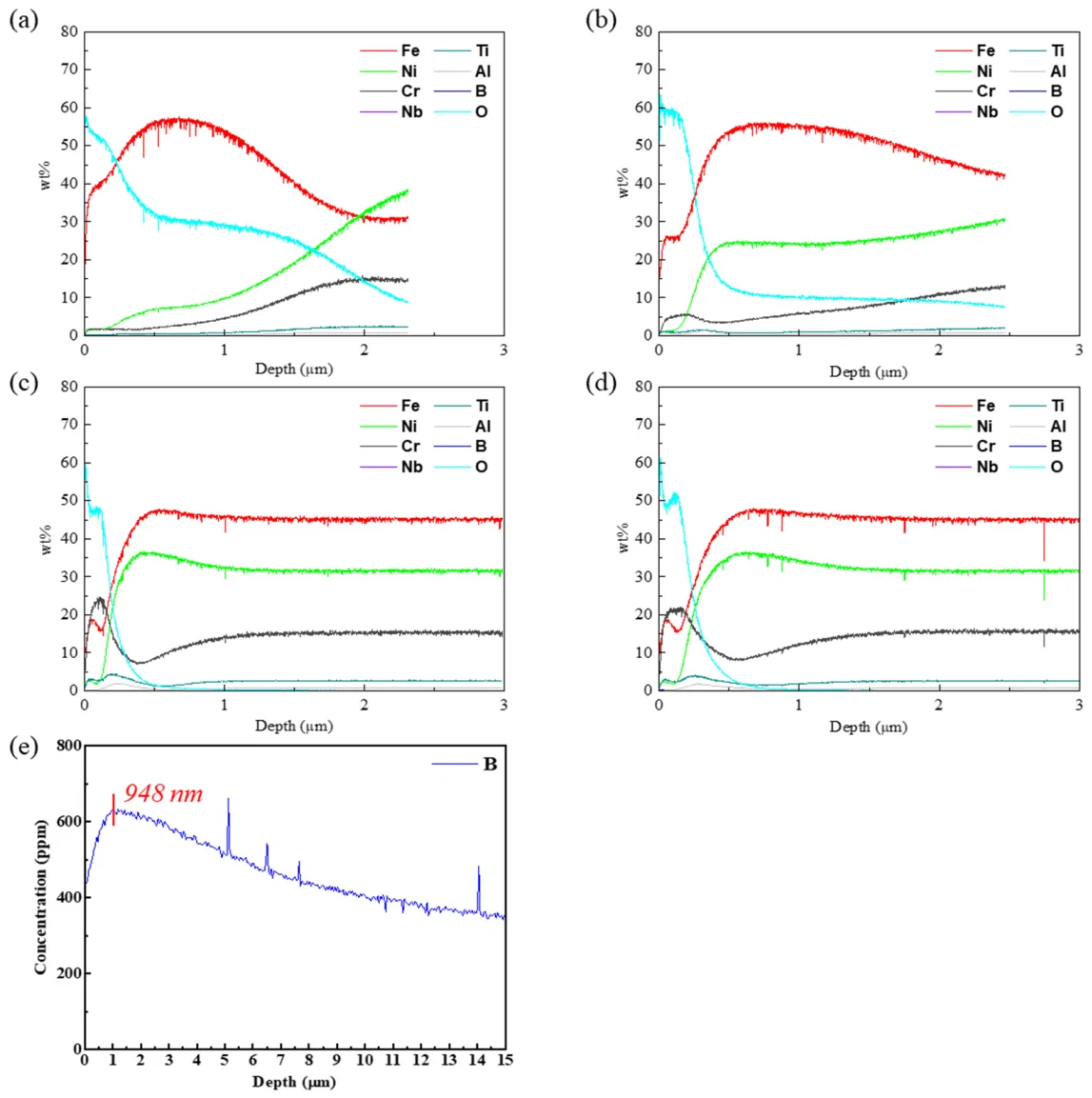
The GD-OES results obtained from the B0 alloy after (**a**) 50 h and (**b**) 100 h of oxidation, and for the B0.1 alloy after (**c**) 50 h and (**d**) 100 h of oxidation. (**e**) The GD-MS results for the B0.1 alloy after 50 h of oxidation.

Because the oxide morphology and TGA/DSC behavior of B0.2 were similar to those of B0.1 after prolonged oxidation, GD-OES and GD-MS analyses were focused on B0.1 as a representative boron-containing alloy to clarify the role of interfacial boron segregation. Figure 7 presents the GD-OES and GD-MS results for the B0 and B0.1 alloys after 50 and 100 h of oxidation. These figures provide a 1D profile of the constituent elements up to a depth of 3 μm. Figure 7a,b show the GD-OES results for the B0 alloy after 50 and 100 h of oxidation, respectively. In the elemental profile after 50 h of oxidation shown in Figure 7a, a high oxygen content layer is observed on the initial surface, followed by a distinct Fe_2_O_3_ layer. As oxidation progresses to 100 h (Figure 7b), the Fe-rich oxide layer thickens, and an increase in Ni within the Fe_2_O_3_ layer was observed. SEM-EDS mapping showed Fe- and Cr-rich regions, while increased Ni enrichment was also observed within the oxide layer after prolonged oxidation. Due to the thick oxide layer in the B0 alloy, the GD-OES results were limited to analyzing an oxidation depth of only 2.25–2.50 μm.

The GD-OES results in Figure 7c,d show little difference between the profiles after 50 and 100 h of oxidation. The GD-MS result in Figure 7e reveals boron enrichment near the oxide/matrix interface. Consistent with the SEM results in Figure 2, the oxide layer, which formed a protective layer with a thickness of approximately 417 nm, remained stable and uniform after 100 h of oxidation. The oxide layer thickness observed in the SEM analysis was in good agreement with the thickness determined from these profiles. As seen in the 1D depth profiles of the B0.1 alloy, the oxide scale consisted predominantly of Fe- and Cr-containing oxides. GD-OES does not directly provide crystallographic phase identification; however, when these elemental profiles are considered together with the XRD and SEM-EDS results, they are consistent with the formation of a thin and continuous spinel-rich Fe-Cr mixed oxide scale. Accordingly, the FeCr_2_O_4_-type assignment is regarded as tentative rather than a definitive stoichiometric identification. Additionally, a Cr depletion zone was observed under the oxide layer (just above the original matrix). The GD-MS results in Figure 7e show boron segregation at the interface between the oxidation layer and the matrix. This indicates the presence of a boron-rich region between the oxide layer and the matrix. These observations suggest a possible association between boron segregation at the oxide/matrix interface and improved oxide-scale stability. Because elemental diffusivity and interfacial adhesion were not directly measured in this study, the influence of boron segregation on elemental transport and interfacial stability is considered a possible interpretation of the observed oxidation behavior rather than a directly demonstrated mechanism.

The present observations are generally consistent with the classical Wagner oxidation theory, in which oxide growth is controlled by ionic diffusion through the oxide scale. In the boron-free alloy, accelerated oxide growth after approximately 75 h was accompanied by the formation of a porous oxide layer and a Cr-depleted region, which is consistent with enhanced outward transport of metallic species during prolonged oxidation. In contrast, both boron-containing alloys maintained a thin and relatively dense oxide layer throughout the oxidation test. Although direct diffusion measurements were not performed, the GD-MS results revealed boron enrichment near the oxide/matrix interface. This interfacial enrichment, together with the observed stability of the oxide scale, suggests a possible influence of boron segregation on elemental transport near the interface. The porous structure observed in the B0 alloy may also be interpreted in terms of the Kirkendall effect, where an imbalance between outward metal diffusion and inward oxygen diffusion leads to vacancy accumulation and pore formation. Such porosity was largely absent in the boron-containing alloys. This difference may be interpreted as reflecting differences in defect and elemental transport near the oxide/matrix interface; however, vacancy behavior was not directly measured in the present study.

According to previous studies, the role of boron in affecting the oxidation resistance of boron-containing superalloys remains unclear. In Co-based superalloys, the addition of 0.04–0.12 at% boron significantly improved oxidation resistance [11]. However, in the Ni-based U720Li alloy, boron penetrated and oxidized within the Cr_2_O_3_ layer during the early stages of oxidation, forming B-rich oxides that reduced the protective properties of Cr_2_O_3_ and accelerated oxidation [13]. The critical distinction appears to be whether boron segregates under the oxide layer or forms B-rich oxide layers within the oxide itself. 

In this study, boron is segregated under the oxide layer without forming a B-rich oxide layer. The results in Figure 7c–e show that the boron segregation is located under the oxide layer. Accelerated oxidation involves elemental transport through the growing oxide scale and may be accompanied by defect accumulation and porosity formation. In the present study, GD-MS revealed boron enrichment near the oxide/matrix interface, while the boron-containing alloys maintained relatively thin and continuous oxide scales. These observations suggest a possible relationship between interfacial boron segregation and oxide-scale stability. However, because elemental diffusivity, vacancy behavior, and interfacial adhesion were not directly measured, the specific role of boron in these processes remains a proposed interpretation and requires further investigation. The combined SEM, EDS, XRD, and depth-profile results indicate distinct oxide-growth behavior between the boron-free and boron-containing alloys. The B0 alloy developed a thick and heterogeneous Fe-rich oxide scale containing Fe_2_O_3_, Cr_2_O_3_, and FeCr_2_O_4_-type spinel contributions. In contrast, the B0.1 and B0.2 alloys maintained thin and continuous spinel-rich Fe–Cr mixed oxide scales. The formation of these continuous Fe–Cr oxide scales is consistent with the reduced oxide-growth rate observed in the boron-containing alloys during prolonged oxidation. However, because of substantial XRD peak overlap and the absence of direct diffusion measurements, the predominance of the FeCr_2_O_4_-type spinel structure and its effect on diffusion should be regarded as a data-consistent interpretation rather than definitive proof. 

Taken together, these observations suggest an association between interfacial boron segregation and improved oxide-scale stability. The possible effects of boron on elemental transport and defect behavior remain to be directly verified. This interfacial stabilization may contribute to improved oxide-scale stability and enhanced oxidation resistance in the boron-containing alloys.

Overall, the present findings are consistent with previous studies emphasizing that the continuity and stability of protective oxide scales play a critical role in determining oxidation resistance [11,13,15]. However, the role of boron appears to depend strongly on its distribution within the oxide scale and at the oxide/matrix interface. Unlike previous studies in which boron formed boron-rich oxides within the oxide scale, no evidence of continuous boron-rich oxide formation was observed in the present FeNi-based alloy. Instead, boron segregation occurred at the oxide/matrix interface, suggesting that the beneficial effect of boron is associated with interfacial stabilization rather than direct participation in oxide formation. This difference highlights the importance of boron distribution in determining the oxidation behavior of boron-containing superalloys.

## 4. Conclusions

This study investigated the effect of boron addition on the oxidation resistance of FeNi-based superalloys. The conclusions are as follows:

1. The present results indicate that boron addition is associated with improved oxidation behavior in FeNi-based superalloys. The observed boron segregation at the oxide/matrix interface is associated with improved oxide-scale stability, while its possible influence on elemental transport remains to be directly verified.
2. Both B0.1 and B0.2 alloys maintained thin and uniform spinel-rich Fe–Cr mixed oxide scales after 100 h of oxidation, with final thicknesses of approximately 417 and 405 nm, respectively. In contrast, the B0 alloy developed a heterogeneous and porous Fe-rich oxide scale with a thickness of approximately 5.41 μm.
3. B0.2 exhibited the thinnest oxide layer at the early oxidation stage (approximately 179 nm after 20 h), suggesting that increased boron content can further suppress initial oxide growth. However, the similar oxide thicknesses of B0.1 and B0.2 after 100 h indicate that the long-term stabilizing effect of boron may become saturated.
4. GD-MS analysis of B0.1 revealed boron segregation at the oxide/matrix interface. The combined results suggest an association between this interfacial segregation and the formation and stability of the thin, continuous Fe–Cr mixed oxide scale observed in the boron-containing alloys. The possible influence of boron on elemental transport and defect behavior remains to be directly verified.

## Figures and Tables

**Table 1:** Chemical composition of FeNi-based superalloys investigated in the present study [16].

Alloy	Fe	Ni	Cr	Nb	Ti	Al	B
B0	38.98	39.21	17.07	1.67	2.03	1.03	-
B0.1	38.90	38.94	17.26	1.68	2.04	1.11	0.08
B0.2	38.7	39.91	16.69	1.74	1.93	0.86	0.17

Note: The compositions are given in atomic percent.

**Table 2:** The thickness of the oxide layer obtained from B0, B0.1, and B0.2 alloys after oxidation for 20 h, 50 h, 100 h, and after creep rupture.

Thickness	20 h	50 h	100 h	After Creep
B0	361 nm	381 nm	5.41 μm	7.90 μm
B0.1	357 nm	374 nm	417 nm	7.87 μm
B0.2	179 nm	321 nm	405 nm	-

**Table 3:** The EDS results of the oxide layer for the B0 alloy under different oxidation time conditions. (For the B0 alloy after 100 h oxidation, three different local regions (A–C) were selected for SEM-EDS point analysis because of the heterogeneous oxide morphology.)

Element	Composition (at.%)				
Oxidation Time (h)	20	50	100		
			A	B	C
O	52.02	52.44	53.85	46.37	6.94
Fe	10.08	11.62	39.15	7.85	24.28
Ni	5.52	8.12	4.8	12.13	61.42
Cr	21.67	21.37	1.68	28.44	3.74
Nb	2.47	1.99	0.04	2.00	0.81
Ti	5.42	1.23	0.31	2.42	1.17
Al	2.82	3.22	0.18	0.78	1.65

**Table 4:** The EDS results of the oxide layer for the B0.1 alloy under different oxidation time conditions.

Element	Composition (at.%)		
Oxidation Time (h)	20	50	100
O	65.36	57.61	48.99
Fe	10.24	21.67	19.46
Ni	7.63	2.83	9.5
Cr	5.46	14.84	16.54
Nb	0.94	0.45	0.57
Ti	5.66	0.74	1.03
Al	4.71	1.85	3.91

**Table 5:** The EDS results of the oxide layer for the B0.2 alloy under different oxidation time conditions.

Element	Composition (at.%)		
Oxidation Time (h)	20	50	100
O	68.91	65.11	70.48
Fe	10.59	17.32	14.53
Ni	2.91	4.89	2.19
Cr	15.33	11.83	10.91
Nb	0.71	0.53	0.41
Ti	0.92	0.00	1.16
Al	0.63	0.33	0.33

**Table 6:** Interval mass-change rates obtained from linear regression of the isothermal TGA data at 1023 K.

Alloy	0–20 h	20–50 h	50–75 h	75–100 h
B0	0.01160	0.00637	0.00352	0.05201
B0.1	−0.00478	0.01527	0.00591	0.00246
B0.2	−0.06737	−0.00935	0.00353	0.00715

Unit: mg cm^−^^2^ h^−1^.

**Table 7:** Time exponent (*n*) values obtained from fitting the isothermal TGA data of the B0, B0.1, and B0.2 alloys at 1023 K.

*n*	0–75 h	75–100 h
B0	0.06262	1.47855
B0.1	0.01254	
B0.2	0.00789	

## Data Availability

The raw data supporting the conclusions of this article will be made available by the authors upon request.

## Abbreviations

The following abbreviations are used in this manuscript:

FE-SEM	Field-emission scanning electron microscopy
EDS	Energy-dispersive X-ray spectroscopy
TGA	Thermogravimetric analysis
DSC	Differential scanning calorimetry
GD-MS	Glow discharge mass spectrometry
GD-OES	Glow discharge optical emission spectroscopy
TD	Transverse direction
